# Clinical course and patient-reported outcomes in conservatively managed spinal cavernous malformations

**DOI:** 10.1007/s00415-026-13715-2

**Published:** 2026-03-08

**Authors:** Abel Clemens Adriaan Sandmann, Marinus Abraham Kempeneers, K. Mariam Slot, René van den Berg, William Peter Vandertop, Dagmar Verbaan, Jonathan M. Coutinho

**Affiliations:** 1 Department of Neurology, Amsterdam UMC, University of Amsterdam, Amsterdam, The Netherlands; 2 Amsterdam Neuroscience, Neurovascular Disorders, Amsterdam, The Netherlands; 3 Department of Neurosurgery, Amsterdam UMC, University of Amsterdam, Amsterdam, The Netherlands; 4 Department of Radiology and Nuclear Medicine, Amsterdam UMC, University of Amsterdam, Amsterdam, The Netherlands

**Keywords:** Cavernous malformation, Spinal cord, Treatment, Conservative management, Natural history, Quality of life

## Abstract

**Background:**

Studies on patients with spinal cavernous malformations (SCM) who were managed conservatively are scarce. We aimed to assess clinical, functional, and patient-reported outcomes in these patients.

**Methods:**

This single-center cohort study included consecutive adult patients with SCM, diagnosed in 1995–2024, who underwent conservative management as the primary treatment strategy and had ≥ 6 months of follow-up. We retrospectively analyzed events of symptomatic hemorrhage (SH) and/or focal neurological deficits (FND) and conducted cross-sectional telephone and questionnaire follow-up. We evaluated functional outcome on the modified Rankin Scale (mRS) and quality of life using EuroQol 5-dimensions 5-levels (EQ-5D-5L) and Patient-Reported Outcome Measurement Information System 29 (PROMIS-29).

**Results:**

We identified 30 patients with SCM, of whom 28 were included (median age 47 years [IQR 36–61], 32% women). Nine (32%) initially presented with SH, 10 (36%) with FND, and 9 (32%) incidentally. During a median follow-up of 6.4 years (IQR 4.0–10.6), 10 (36%) patients experienced SH/FND and 5 (18%) underwent surgical intervention. The annual rate of SH/FND was 5.1% (95% CI 2.5–9.4%). At final follow-up, 26 (93%) patients completed the questionnaire and 16 (57%) were functionally independent (mRS ≤ 2). Patients had lower utility-weighted EQ index scores than the general population (0.63 versus 0.87, *p* < 0.001) and reported more PROMIS-29 anxiety/fear (56.8 versus 50.3, *p* = 0.002), depression/sadness (55.9 versus 50.3, *p* = 0.023), and fatigue (55.2 versus 49.4, *p* = 0.029).

**Conclusions:**

Among 28 conservatively managed patients with SCM, 23 (82%) did not require surgical treatment during follow-up and the majority remained functionally independent. However, patients do report worse health than the general population, particularly mental health.

**Supplementary Information:**

The online version contains supplementary material available at 10.1007/s00415-026-13715-2.

## Introduction

Spinal cavernous malformations (SCM) are intradural, intramedullary vascular malformations that can cause symptomatic hemorrhage (SH) and non-hemorrhagic focal neurological deficits (FND) [1]. Episodes of hemorrhage or expanding SCMs may rapidly precipitate neurological symptoms owing to the spinal cord’s eloquence and confined anatomical space [2]. The majority of SCMs are solitary and sporadic, while familial cases typically feature multiple lesions, including cerebral cavernous malformations (CCM) [3]. Although SCMs are rare, representing around 5% of the cavernous malformations in the central nervous system [4], the number of published cases has increased since the widespread availability of MRI [5, 6].

Treatment strategies for SCM include surgical resection and conservative management. In a meta-analysis of studies on the treatment of SCM, 90% of cases were treated surgically, and outcomes were better after surgical treatment than with conservative management [7]. However, direct comparisons of the treatment groups were complicated by a lack of baseline characteristics stratified by treatment strategy, methodological limitations in the included studies, and the frequent lack of a control group. Additionally, since the clinical course of SCM is not fully understood, observed improvements might have occurred independently of intervention, reflecting natural recovery from symptomatic events.

To better understand the clinical course of SCM, studies are needed that determine the outcomes of a relatively unselected cohort of patients with SCM who are managed conservatively. Moreover, although patient-centered evidence is important for informing patient counseling, studies incorporating patient-reported outcome measures (PROMs) are scarce, particularly in patients with conservatively managed SCMs. At our hospital, we predominantly adopt conservative management as the primary approach for patients with SCM, enabling a systematic evaluation of this treatment strategy. Therefore, the objective of this study was to analyze the clinical course and quality of life in patients with SCM who underwent conservative management as the primary treatment strategy.

## Methods

### Design and patient selection

In this retrospective, single-center cohort study, we included consecutive patients with SCM diagnosed at our hospital between January 1995 and December 2024. We excluded patients who underwent surgical treatment as a primary treatment strategy. Patients who initially underwent conservative management but were subsequently treated surgically were included. Further exclusion criteria were age < 18 years and < 6 months of follow-up. Patients were identified from our ongoing prospective neurovascular registry and the hospital medical records system using International Classification of Diseases (ICD) 10 codes [8]. Each diagnosis of SCM was verified on the diagnostic MR images by certified neuroradiologists.

The Institutional Review Board of the Amsterdam UMC waived the need for ethical approval due to the study’s observational design (W22_196 # 22.244). As previously described [9], we first conducted an opt-out procedure for the reuse of clinical data and subsequently obtained informed consent for patients who participated in the questionnaire. This report complies with the STROBE guidelines for observational research [10].

### Data collection and outcomes

Baseline characteristics and data related to follow-up and study outcomes were extracted from the hospital medical records and subsequently verified during systematic telephone follow-up conducted by the first author, inviting all patients to participate in a structured questionnaire (Online Resource 1). If there was a discrepancy between the records and the questionnaire or in case of missing data, we requested documentation from the referring hospital or general practitioner. They were also approached to request updated contact information if a patient could not be reached. After three unsuccessful call attempts, we sent an email invitation with the study information and a survey link. Participants who showed interest but did not return the questionnaire within 2 weeks received one follow-up reminder via telephone. Completion of the questionnaire marked the endpoint of follow-up.

Baseline was defined as the initial presentation, marked by the onset of symptoms or, in asymptomatic cases, the medical consultation from which follow-up was available that resulted in the diagnosis of SCM. Baseline characteristics included demographics, mode of presentation (SH, FND, or incidental), family history, genetic testing (if applicable), and lesion characteristics (number, location, and size of SCM, and presence of CCMs). Among symptomatic patients, we classified the clinical course as acute (sudden onset of symptoms with diagnosis within 1 week) or progressive (worsening of symptoms over time with diagnosis more than 1 week after the symptom onset). We also noted the type of symptoms (e.g. motor or sensory). Cases were considered familial if confirmed by genetic testing, if there were at least five cavernous malformations, or if a first-degree family member had at least one lesion [9]. Location of the SCM was assessed at the spinal cord level and in the horizontal plane. Horizontal locations were classified as either ventral or dorsal, and either deep or superficial, with superficial SCMs being in contact with the pial surface on MRI, whereas deep lesions were fully embedded in the spinal cord [11, 12]. Size was measured as the diameter of the (largest) SCM on axial T2-weighted MRI sequences.

Data on follow-up and study outcomes included the occurrence of clinical events (i.e. SH or FND) and surgical intervention. For patients undergoing surgery during follow-up, we noted the reason for and outcome of treatment. Events of SH and FND were distinguished based on whether timely radiological imaging was available, showing recent hemorrhage. The main study outcome was a composite of SH or FND (SH/FND), as some FND might be undetected hemorrhages [13]. We also quantified SH alone for additional comparisons. Functional outcomes at final follow-up were assessed by the first author on the modified Rankin Scale (mRS), ranging from 0 (no symptoms) to 6 (death) [14], with scores of ≤ 2 being classified as functionally independent. We used the questionnaire in Online Resource 1 if available, or alternatively telephone consultation or review of hospital medical records.

In addition to clinical and functional outcomes, we collected data on quality of life using the PROMs included in the questionnaire. EQ-5D-5L (EuroQol five-dimensions five-levels) assesses health across five domains (mobility, self-care, usual activities, pain/discomfort, and anxiety/depression) in which the respondent selects one of five levels (1 no problems, 2 slight problems, 3 moderate problems, 4 severe problems, or 5 extreme problems), based on their condition at the time of completion [15]. Additionally, it includes the EQ visual analog scale (EQ-VAS), a rating of overall health between 0 and 100 (from worst to best imaginable health). PROMIS-29 (Patient-Reported Outcome Measurement Information System) evaluates health in the previous 7 days and contains four questions for each of the seven domains (physical function, anxiety/fear, depression/sadness, fatigue, sleep disturbance, ability to participate in social roles/activities, and pain interference) [16]. Responses to these 28 items are scored on a five-point scale (1–5), corresponding to excellent versus poor health, whereas question 29 is a separate numeric rating item for pain intensity on a scale of 0–10.

### Statistical analysis

Data are mostly presented descriptively. We calculated annual rates of SH/FND, in which follow-up was censored at the time of the first event. Moreover, we performed Kaplan–Meier time-to-event analyses for the occurrence during follow-up of SH/FND, with calculation of cumulative rates at 5 years and 10 years. Additionally, we performed sensitivity analysis of SH alone, calculating annual rates and Kaplan–Meier cumulative rates. Furthermore, these annual and cumulative rates were calculated for all patients in the study population, and stratified by mode of initial presentation (SH/FND versus incidentally, or SH versus FND or incidentally). For comparisons of annual rates, we used the Chi-squared test [17].

The EQ-5D-5L data were converted into utility-weighted EQ index scores to assess each participant’s overall health status. Each participant was assigned a five-digit health code (e.g. 55545 or 12354), which corresponded to their responses across the five domains. These codes were then translated into domain-specific weights using the EQ-5D-5L Dutch tariff (*n* = 1003) [18]. Subsequently, we subtracted these weights from 1, yielding values between 1 (indicating full health) and 0 (representing death), and negative scores reflecting health states worse than death. The mean utility-weighted EQ index score was compared using the unpaired *t*-test with that of a Dutch reference population (*n* = 979, in whom the mean score was 0.87, standard deviation [SD] ± 0.17) [18], as well as between patients who underwent surgical treatment during follow-up and those who were managed conservatively throughout.

The PROMIS-29 responses were summed into domain sum scores ranging from 4 to 20. The sum scores were then converted into domain-level *T* scores, standardized against a reference population with a mean of 50 and a SD of 10. This conversion was conducted using the HealthMeasures Scoring Service, which applies response pattern scoring, generating the most accurate scores for PROMIS instruments [19]. This method incorporates all completed responses and item parameters, based on the item response theory [20]. We oriented all domain-level *T* scores in one direction, with higher values corresponding to poorer health outcomes. Distributions of domain T scores were compared using the Mann–Whitney *U* test with those of Dutch reference populations, selected to be representative of the general population in terms of sociodemographic characteristics (*n* > 1000) [21–24]. The median (interquartile range [IQR]) *T* scores of Dutch reference populations were as follows: physical function 49.5 (42.8–57.7), anxiety/fear 50.3 (42.4–57.0), depression/sadness 50.3 (42.2–56.7), fatigue 49.4 (40.8–57.2), sleep disturbance 49.9 (42.4–57.0), social participation 49.2 (43.6–56.2), and pain interference 55.6 (49.4–60.9). We also compared domain *T* scores between patients who underwent surgical treatment during follow-up and those managed conservatively throughout. Analyses were done using IBM SPSS Statistics version 28 [25], and MedCalc Statistical Software version 22 [26].

## Results

We identified 30 patients with SCM (12 through the registry and 18 via ICD-10 codes). Two patients were excluded as they opted out (*n* = 1) or underwent surgical intervention as the primary treatment strategy (*n* = 1, details in Online Resource 2). Among 28 included patients, the median age at initial presentation was 47 years (IQR 36–61) and 9 (32%) were women (Table 1). Nine (32%) patients presented with SH, 10 (36%) with FND, and 9 (32%) incidentally. Among symptomatic patients, the clinical course was acute in 9 (47%) and progressive in 10 (53%), while 14 (74%) had motor symptoms, 18 (95%) sensory symptoms, 16 (84%) pain, 7 (37%) bladder and/or bowel symptoms, and 12 (63%) gait disturbance. Details about why conservative management was the preferred primary strategy in these patients are provided in Online Resource 3.

**Table 1:** Baseline characteristics of all patients and stratified by mode of presentation

Variable	All patients (*n* = 28)	Presentation with SH/FND (*n* = 19)	Incidental presentation (*n* = 9)
Age (years)	47 (36–61)	52 (32–61)	45 (38–61)
Sex (female)	9 (32%)	7 (37%)	2 (22%)
Mode of presentation			
SH	9 (32%)	9 (47%)	–
FND	10 (36%)	10 (53%)	–
Incidental	9 (32%)	–	9 (100%)
Familial^a^	11 (39%)	4 (21%)	7 (78%)
Multiple SCMs	4 (14%)	3 (16%)	1 (11%)
Concomitant CCMs	12 (43%)	5 (26%)	7 (78%)
Genetically confirmed	5 (18%)	2 (11%)	3 (33%)
CCM1	4 (14%)	1 (5%)	3 (33%)
CCM2	1 (4%)	1 (5%)	0 (0%)
CCM3	0 (0%)	0 (0%)	0 (0%)
Location of SCM			
At spinal cord^b^			
Cervical	9 (28%)	5 (23%)	4 (40%)
Thoracic	21 (66%)	15 (68%)	6 (60%)
Lumbar	2 (6%)	2 (9%)	0 (0%)
In horizontal plane^c^			
Dorsal (versus ventral)	17 (61%)	12 (63%)	5 (56%)
Superficial (versus deep)^d^	21 (75%)	16 (84%)	5 (56%)
Diameter of SCM (mm)^c^	7 (4–11)	7 (4–10)	6 (3–16)

Data are median (IQR) or number (%)

*CCM* cerebral cavernous malformation, *FND* non-hemorrhagic focal neurological deficit, *IQR* interquartile range, *SCM* spinal cavernous malformation, *SH* symptomatic hemorrhage

^a^Cases were considered familial if a genetic mutation was detected, if there were at least five cavernous malformations, or if a first-degree family member had at least one lesion

^b^Numbers do not add up to 28 patients, but to 32 lesions as four patients had two SCMs

^c^Largest lesion was used if there were multiple SCMs

^d^Superficial SCMs were in contact with the pial surface on MRI, while deep lesions were fully embedded in the spinal cord

Patients were followed for 276.4 person-years (median 6.4 years [IQR 4.0–10.6]). After completion of the opt-out procedure for all patients, 26 (93%) participated in the questionnaire. The other two patients were consulted via telephone. One of them was unable to participate in the questionnaire due to a poor health state and died later because of aspiration, unrelated to SCM. The other declined to participate in a quality-of-life questionnaire. Both patients continued their consent to the use of clinical data. Baseline data and mortality status were available for all patients, and clinical follow-up was complete. Data were verified through the questionnaire for all but two patients.

### Clinical and functional outcomes

**Fig. 1:**
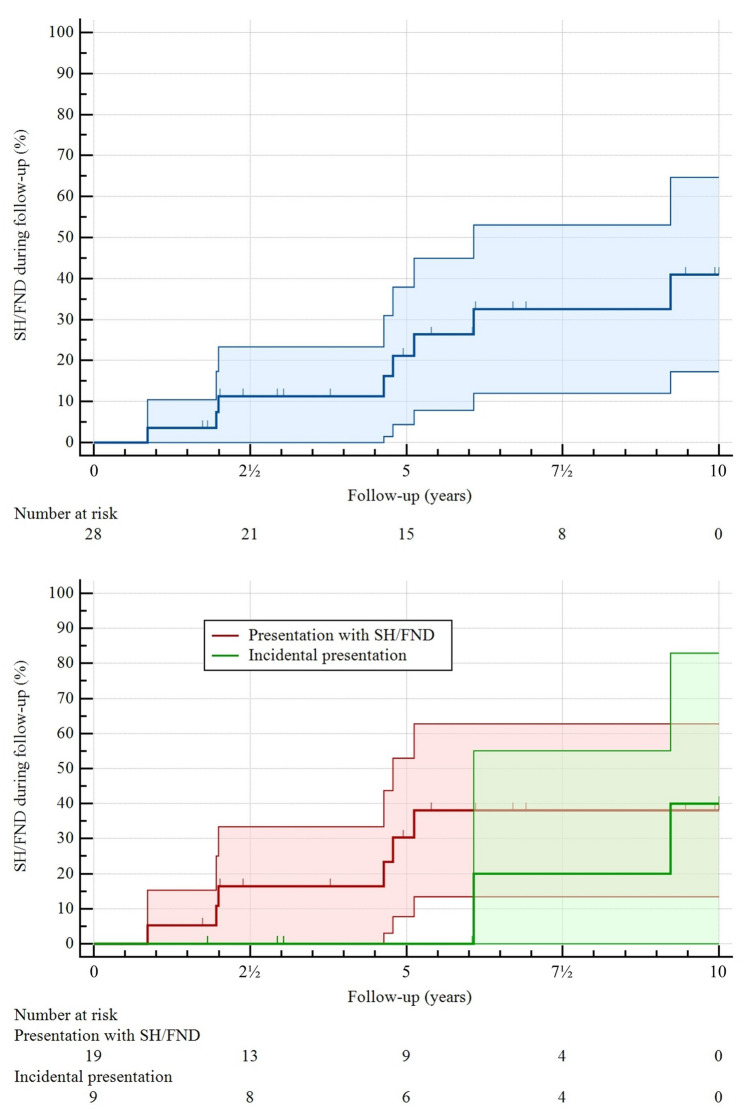
Kaplan–Meier analysis for the progression to SH/FND during follow-up among all patients in the study population (blue) and stratified by presentation with SH/FND (red) versus incidentally (green); SH/FND, symptomatic hemorrhage or non-hemorrhagic focal neurological deficit

During follow-up, 10 (36%) patients experienced SH/FND (*n* = 7 SH and *n* = 5 FND [*n* = 2 both], Table 2). Three (11%) patients had multiple events (*n* = 2 two events, *n* = 1 five events). The annual rate of SH/FND was 5.1% (95% CI 2.5–9.4%), with cumulative 5-year and 10-year rates of 21% (95% CI 4–38%) and 41% (95% CI 17–65%), respectively (Fig. 1). In sensitivity analysis of SH alone, the annual rate was 3.4% (95% CI 1.4–7.0%), with cumulative rates in Online Resources 4 and 5. Five (18%) patients underwent surgical treatment (*n* = 3 due to recurrent SH [or FND], *n* = 2 because of the patient’s preference, further details in Online Resource 2), a median of 24 months (IQR 22–137) after the initial presentation. At final follow-up, 16 (57%) patients were functionally independent, with similar rates between patients who underwent surgery during follow-up and those managed conservatively throughout (3/5 [60%] versus 13/23 [57%]).

**Table 2:** Outcomes during follow-up

Variable	All patients (*n* = 28)
Follow-up (years)	6.4 (4.0–10.6)
SH/FND	10 (36%)
Annual rate of SH/FND	5.1% (2.5–9.4%)
Presentation with SH/FND (*n* = 19)	6.3% (2.5–13.0%)
Incidental presentation (*n* = 9)	3.5% (0.7–10.3%)
Cumulative 5-year rate of SH/FND	21% (4–38%)
Presentation with SH/FND (*n* = 19)	30% (8–53%)
Incidental presentation (*n* = 9)	0% (0–0%)
Cumulative 10-year rate of SH/FND	41% (17–65%)
Presentation with SH/FND (*n* = 19)	38% (13–63%)
Incidental presentation (*n* = 9)	40% (0–83%)
SH	7 (25%)
Annual rate of SH	3.4% (1.4–7.0%)
Presentation with SH (*n* = 9)	8.4% (1.7–24.4%)
Presentation with FND or incidentally (*n* = 19)	2.4% (0.6–6.0%)
FND	5 (18%)
Surgical intervention	5 (18%)
Complete resection	4 (14%)
Partial resection	1 (4%)
mRS score at final follow-up	
0	4 (14%)
1	4 (14%)
2	8 (29%)
3	5 (18%)
4	6 (21%)
5	0 (0%)
6	1 (4%)

Data are median (IQR), number (%), or rate (95% CI); cumulative 5-year and 10-year rates of SH alone in Online Resource 4

*CI* confidence interval, *FND* non-hemorrhagic focal neurological deficit, *IQR* interquartile range, *mRS* modified Rankin Scale, *SH* symptomatic hemorrhage

The annual rate of SH/FND during follow-up in patients who initially presented with SH/FND was 6.3% (95% CI 2.5–13.0%) and in those who presented incidentally 3.5% (95% CI 0.7–10.3%), which did not differ significantly (*p* = 0.39). Cumulative rates are shown in Fig. 1 and Table 2. In sensitivity analysis of SH alone, the annual rate tended to be higher in patients who initially presented with SH (8.4% [95% CI 1.7–24.4%]) than in those who presented with FND or incidentally (2.4% [95% CI 0.6–6.0%], *p* = 0.08). Cumulative rates of SH alone are provided in Online Resources 4 and 5. Proportions of patients who were functionally independent at final follow-up were comparable between patients who initially presented with SH/FND (11/19 [58%]) and those incidentally (5/9 [56%]).

### Patient-reported outcomes

**Fig. 2:**
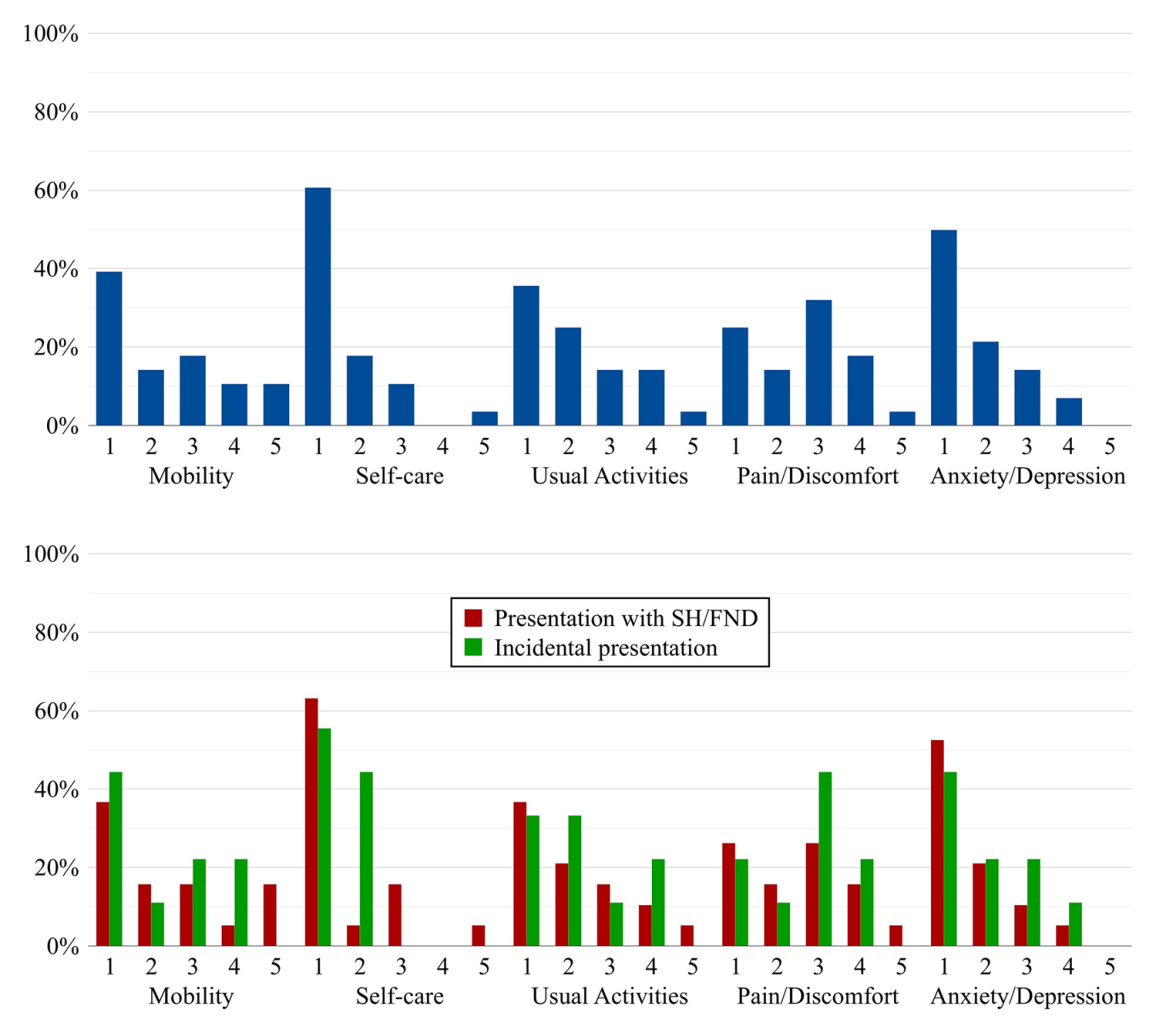
Bar graphs of EQ-5D-5L distributions of all patients in the study population (blue) and stratified by presentation with SH/FND (red) versus incidentally (green); 1, no problems; 2, slight problems; 3, moderate problems; 4, severe problems; 5, extreme problems; SH/FND, symptomatic hemorrhage or non-hemorrhagic focal neurological deficit

Distributions of EQ-5D-5L levels are shown in Fig. 2. The mean EQ-VAS score was 67 (SD ± 19). The mean utility-weighted EQ index score was 0.63 (SD ± 0.31), which was lower compared to the 0.87 (SD ± 0.17) of the Dutch reference population (*p* < 0.001). Mean utility-weighted EQ index scores were similar between patients who underwent surgical treatment during follow-up and those managed conservatively throughout (0.75 [SD ± 0.28] versus 0.60 [SD ± 0.32], respectively, *p* = 0.39).

**Fig. 3:**
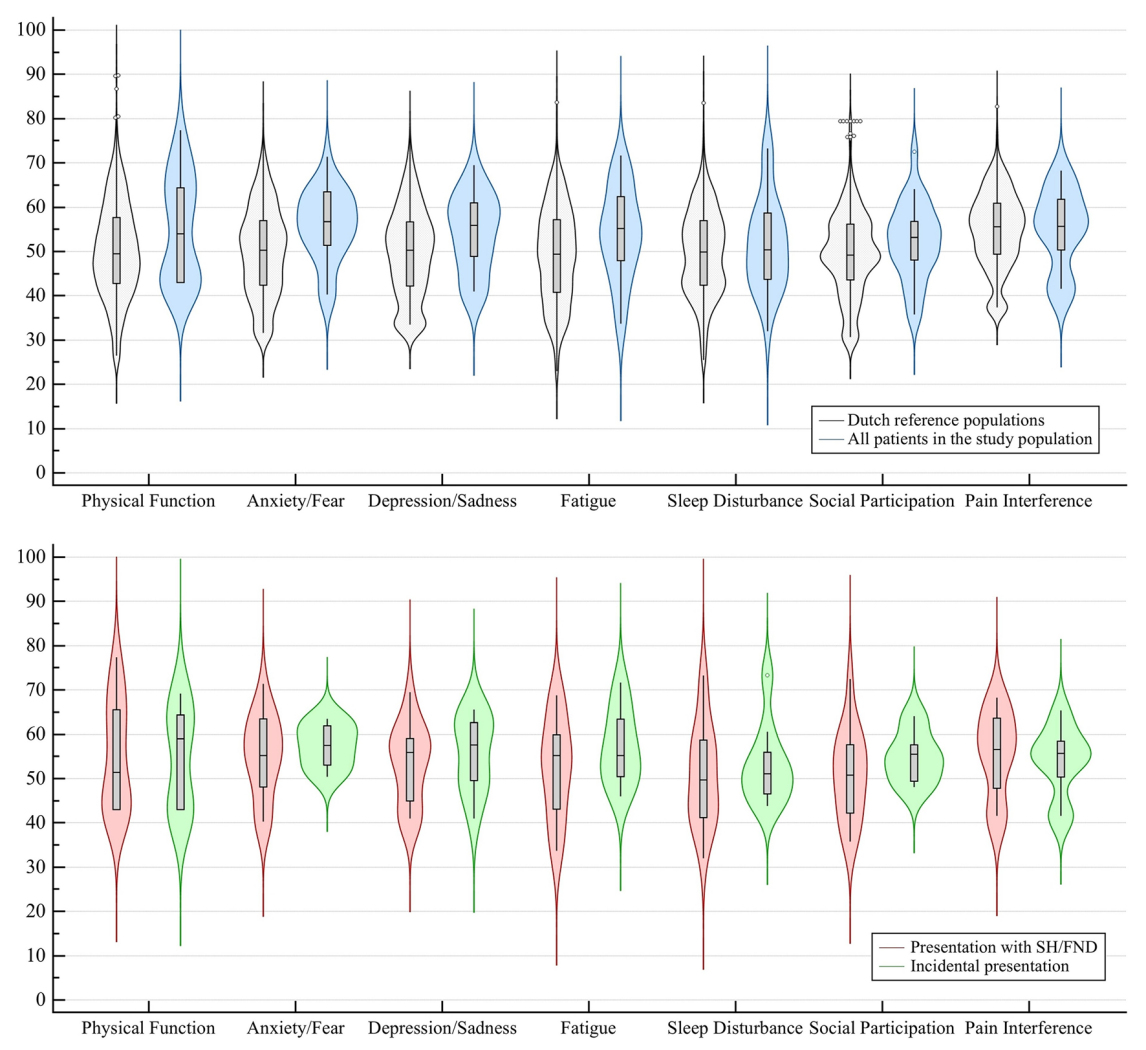
Violin plots of PROMIS-29 domain T score distributions of Dutch reference populations (black) versus all patients in the study population (blue) and stratified by presentation with SH/FND (red) versus incidentally (green); higher *T* scores indicate worse outcomes; boxes are IQRs, horizontal lines in boxes are medians, whiskers are the 95% CI around the median, dots are outliers; *CI* confidence interval, *IQR* interquartile range, *SH/FND* symptomatic hemorrhage or non-hemorrhagic focal neurological deficit

Distributions of PROMIS-29 domain *T* scores, as compared to those of Dutch reference populations, were comparable for physical function (54.0 [IQR 43.0–65.3] versus 49.5), sleep disturbance (50.4 [IQR 43.7–58.7] versus 49.9), social participation (53.2 [IQR 48.1–57.7] versus 49.2), and pain interference (55.7 [IQR 47.5–62.3] versus 55.6). The domain *T* score distributions were, however, worse for anxiety/fear (56.8 [IQR 51.2–63.5] versus 50.3, *p* = 0.002), depression/sadness (55.9 [IQR 48.9–61.4] versus 50.3, *p* = 0.023), and fatigue (55.2 [IQR 47.3–62.5] versus 49.4, *p* = 0.029). There were no significant differences between the domain *T* score distributions of patients who were treated surgically during follow-up and those managed conservatively throughout (Online Resource 6). The median PROMIS-29 pain intensity score was 5 (IQR 2–7). Sociodemographic characteristics of the study cohort, Dutch reference populations, and the Dutch adult population were comparable (Online Resource 7) (Fig. 3).

EQ-5D-5L level distributions stratified by initial presentation with SH/FND versus incidentally were comparable across all domains, though extreme problems in any domain occurred only in patients who initially presented with SH/FND (Fig. 2). Similarly, PROMIS-29 domain *T* distributions score were also comparable between patients who initially presented with SH/FND versus incidentally (Fig. 3).

## Discussion

In this single-center cohort study, we evaluated conservative management as the primary treatment strategy for patients with SCM. We found that within 5 years of follow-up, approximately one in five patients experienced SH or FND, with an annual rate of 5.1% per person-year. The rates of SH or FND seemed higher after an initial presentation with the same symptoms compared to other presentations, though the sample size was too small to detect statistically significant differences. Among primarily conservatively managed patients, 5/28 (18%) required surgical treatment during follow-up. At final follow-up, most patients remained functionally independent, but patients reported worse overall health compared to the general population, with affected domains being anxiety/fear, depression/sadness, and fatigue.

In the current literature, the majority of published cases of SCM have been treated surgically, with many studies lacking a control group of patients who underwent conservative management [7]. Although some direct comparative studies exist, they often vary in their analysis of outcomes according to the treatment strategy assigned at diagnosis or the treatment eventually performed [11, 12, 27–29]. A limited number of studies included an analysis of conservatively managed SCMs [30–32], in one of which 35% of *n* = 71 patients were treated surgically [32]. In another comparative study (*n* = 85), after excluding 25% of patients who received up-front surgical treatment, 17% of the remaining conservatively managed patients with SCM underwent surgery during follow-up [28]. This is comparable to the rate of 18% at our institution, although we treated only one patient surgically directly after diagnosis (4%), in line with our policy to primarily treat conservatively. This suggests that intervention during a conservative strategy is often unnecessary, which may be reassuring when considering this treatment as the primary approach, although it remains uncertain whether outcomes of conservative management are superior as compared to if early resection would have been performed. Furthermore, as with the surgically treated patients in our study, previous literature demonstrates that those who undergo surgical treatment typically have dorsal or superficial lesions, while ventral or deep SCMs are more frequently left untreated due to risks associated with resection [33]. In contrast, the conservatively managed patients in our study mostly had dorsal and/or superficial SCMs, providing outcomes of potentially surgically accessible lesions under conservative management.

The most significant risk associated with untreated SCMs is SH, as it may impair spinal cord function and lead to disability. The per-patient annual rate of hemorrhage from SCM in a study that focused on conservative management was 4.7% (*n* = 71, of whom *n* = 44 initially presented with a hemorrhage, mean follow-up 3.6 years) [32]. Additional bleeding rates were estimated in two comparative studies, reporting annual rates of 3.9–5.5% [11, 28]. However, the lower value was calculated among 27 patients who were managed conservatively throughout the entire follow-up period (mean 2.8 years, all initially presented with hemorrhage), excluding those treated surgically at baseline or during follow-up [11], whereas surgical intervention is often performed following a SH. Moreover, the upper value was calculated in 64 patients (*n* = 37 hemorrhagic) who were managed conservatively as the primary strategy, of whom 11 underwent surgery during follow-up (mean 4.5 years), while primarily surgically treated patients were excluded [28]. These rates were slightly higher than in our study (3.4%), possibly because they often reflected rates of recurrent hemorrhage, as many patients had initially presented with hemorrhage (compared to only 32% in our study), although wide confidence intervals due to small sample sizes should be acknowledged. Additionally, those studies had shorter average follow-up than our study, which may have inflated the risks, as risks may be increased shortly after a hemorrhage, a pattern recently observed in patients with CCM [34]. Furthermore, bleeding rates from SCM (3.4–5.5%) might be slightly higher than from CCM (1.3–3.7%) [9, 34, 35], but further research is needed to reach definitive conclusions.

Quality of life in patients with SCM has previously been assessed only once, in a study that included patients with cavernous malformations throughout the entire central nervous system, without separately analyzing the nine patients with SCM [36]. In the current study, we found that the overall health of patients with SCM was reduced compared to the Dutch reference population (utility-weighted EQ index scores 0.63 versus 0.87). We were unable to detect differences between initial presentations with SH/FND and those incidentally, which may be due to the low numbers in this stratified analysis. In the overall study population, patients predominantly reported fatigue and problems in mental domains. Physical domains, however, were largely comparable to the general population, which may indicate physical recovery from symptomatic events, although the small sample size in our study limited the ability to detect statistically significant differences. Nonetheless, this may suggest that mental health requires particular attention to improve the overall quality of life among these patients, as was suggested for patients with CCM [37]. Furthermore, patients with SCM reported worse overall health than patients with CCM (utility-weighted EQ index scores 0.63 versus 0.79) [37]. This is in accordance with the functional independence proportions for patients with SCM (57%) versus CCM (77%) [9]. In the spinal cord, even small hemorrhages or lesion expansions may lead to symptoms or disability, whereas CCMs can occur in non-eloquent areas where limited rupture may remain clinically silent.

This study provides clinical and novel patient-reported outcomes with a unique focus on conservative management as the primary strategy for patients with SCM, a treatment modality underrepresented in prior research. Despite the rarity of SCMs, we captured a substantial follow-up duration in person-years and included a consecutive patient cohort, reflecting real-world practice where surgical intervention may occasionally be required during a conservative strategy. Moreover, the response rate to the questionnaire was high, which allowed us to verify data for all but two patients. Furthermore, we reported functional outcomes and assessed quality of life specifically in patients with SCM, offering insights that may help clinicians identify domains with the highest potential for improvement of the outcomes of these patients. Several limitations should also be acknowledged. First, this study was conducted at a single tertiary care center, which may have led to overestimation of event rates, although patients with SCM are generally referred to such centers for specialized care or diagnostic confirmation. Second, the retrospective design may have introduced selection and information biases. Third, the sample size was small, which resulted in limited statistical power and a low number of events, preventing multivariable analyses to adjust for potential confounders. Fourth, some cases of SCM that also harbored CCMs overlapped with the cases of studies on CCM [9, 37], complicating the comparison of functional and patient-reported outcomes, which are patient-specific rather than lesion-specific. Fifth, because of the small number of patients, we decided not to compare sporadic and familial cases, while the proportion of patients with the familial form was relatively high in our study.

This study of conservatively managed patients with SCM shows that more than four out of five patients do not require surgical intervention during follow-up. Under conservative management, the annual rate of SH/FND is approximately 5%, and initial presentation with the same symptoms may lead to higher rates. These rates can be weighed against the estimated up-front risks of surgery, though they should not be extrapolated to patients’ lifetimes, as risks may change over time. Patients with conservatively managed SCMs may report reduced quality of life compared with the general population, largely due to impaired mental health, underscoring an important area that warrants attention in clinical practice. Future research could assess functional status longitudinally at prespecified time points to monitor potential recovery, and researchers should aim for larger sample sizes, ideally via international, multicenter collaborations or using patient registries. Such studies should be controlled to compare the treatment strategies, while ensuring baseline comparability, to ultimately determine the optimal treatment for patients with SCM.

## Supplementary Information

Below is the link to the electronic supplementary material.

Supplementary file1 (DOCX 280 KB)

Supplementary file2 (PDF 296 KB)

## Data Availability

Anonymized data supporting the findings of this study will be made available for scientific research on reasonable request to the corresponding author.
