# Supplementary material for: Clinical course and patient-reported outcomes in conservatively managed spinal cavernous malformations
Source: J Neurol. 2026 Mar 8;273(3):188. doi: 10.1007/s00415-026-13715-2 (PMC12968117; doi:10.1007/s00415-026-13715-2)
Supplement: Supplementary file 1 — Supplementary file1 (DOCX 280 KB) [file 415_2026_13715_MOESM1_ESM.docx]

**Supplementary Material**

**Title:** Clinical course and patient-reported outcomes in conservatively managed spinal cavernous malformations

**Journal:** Journal of Neurology

**Authors:** Abel Clemens Adriaan Sandmann, Marinus Abraham Kempeneers, K. Mariam Slot, René van den Berg, William Peter Vandertop, Dagmar Verbaan, and Jonathan M. Coutinho

**Corresponding author:** Jonathan Coutinho, Amsterdam UMC, University of Amsterdam, Department of Neurology, Amsterdam, The Netherlands, j.coutinho@amsterdamumc.nl

Online Resource 1. Questionnaire (in separate file)

Online Resource 2. Details of six patients who underwent surgical intervention as the primary treatment strategy or during follow-up after an initial conservative strategy

| **Age^a^** | **Sex** | **Mode of presentation** | **Location of SCM** | **Primary treatment strategy** | **Time^b^** | **Reason for and outcome of intervention** |
| --- | --- | --- | --- | --- | --- | --- |
| 67 | F | SH | Dorsal & superficial | Surgical treatment (excluded) | 0 | First SH, progressive symptoms, urinary retention, and complete paralysis; treated four days after diagnosis; postoperative persistent symptoms; mRS 4 |
| 37 | M | SH | Dorsal & superficial | Conservative management | 24 | Recurrent SH and mRS 5; treatment complicated by respiratory failure, urgent re-intubation and ICU admission; improvement after second operation; mRS 6 (unrelated) |
| 14 | M | SH | Dorsal & superficial | Conservative management | 24 | Recurrent SH and mRS 5; postoperative cauda equina syndrome, later substantial improvement; mRS 0 |
| 24 | M | FND | Ventral & superficial | Conservative management | 11 | Recurrent FND, mRS 1, patient’s preference; treated in other center, partial resection; postoperative pain, paresis, and cervical instability; mRS 2 |
| 36 | F | Incidental | Dorsal & deep | Conservative management | 241 | Fourth SH/FND and mRS 5; postoperative worsening, later improvement; mRS 4 |
| 23 | M | SH | Dorsal & superficial | Conservative management | 8 | First FND, progressive symptoms, mRS 2; patient’s preference; postoperative improvement; mRS 1 |

^a^Age at initial presentation in years; ^b^Time from diagnosis to intervention in months; F, female; FND, non-hemorrhagic focal neurological deficit; ICU, intensive care unit; M, male; mRS, modified Rankin Scale; SCM, spinal cavernous malformation; SH, symptomatic hemorrhage.

Online Resource 3. Details of symptomatic patients who underwent conservative management as the primary treatment strategy

| **Age^a^** | **Sex** | **Mode of presentation** | **Location of SCM** | **Clinical course** | **Reason for conservative management** |
| --- | --- | --- | --- | --- | --- |
| 65 | M | FND | Ventral & superficial | Progressive | Relation between sensory disturbance and SCM uncertain |
| 37 | M | SH | Dorsal & superficial | Acute | Spontaneous recovery expected after hematoma resorption |
| 36 | M | FND | Dorsal & superficial | Acute | Symptoms due to edema that were expected to resolve |
| 52 | F | FND | Dorsal & deep | Acute | Improvement of symptoms from deep SCM |
| 29 | F | SH | Ventral & superficial | Acute | Higher estimated risk of surgery due to anterior location |
| 50 | M | FND | Dorsal & superficial | Acute | Improvement of symptoms and higher estimated risk of surgery |
| 57 | F | FND | Dorsal & superficial | Progressive | Mild symptoms and higher estimated risk of surgery |
| 14 | M | SH | Dorsal & superficial | Acute | Improvement of symptoms after hematoma resorption |
| 24 | M | FND | Ventral & superficial | Progressive | Second opinion; higher estimated risk of surgery due to anterior location |
| 61 | M | FND | Dorsal & superficial | Progressive | Mild symptoms and higher estimated risk of surgery |
| 52 | F | SH | Dorsal & superficial | Acute | Diagnosis confirmed after hematoma resorption when symptoms had improved |
| 64 | M | FND | Ventral & deep | Progressive | Higher estimated risk of surgery due to anterior and deep location |
| 32 | M | SH | Dorsal & superficial | Progressive | Mild symptoms and higher estimated risk of surgery |
| 55 | F | FND | Dorsal & superficial | Progressive | Relation between symptoms and SCM uncertain |
| 23 | M | SH | Dorsal & superficial | Progressive | Spontaneous recovery expected after hematoma resorption |
| 73 | M | FND | Ventral & superficial | Progressive | Higher estimated risk of surgery due to anterior location |
| 38 | F | SH | Dorsal & deep | Acute | Spontaneous recovery expected after hematoma resorption |
| 75 | M | SH | Ventral & superficial | Progressive | Higher estimated risk of surgery due to anterior location |
| 60 | F | SH | Ventral & superficial | Acute | Higher estimated risk of surgery due to anterior location |

^a^Age at initial presentation in years; F, female; FND, non-hemorrhagic focal neurological deficit; M, male; SCM, spinal cavernous malformation; SH, symptomatic hemorrhage.


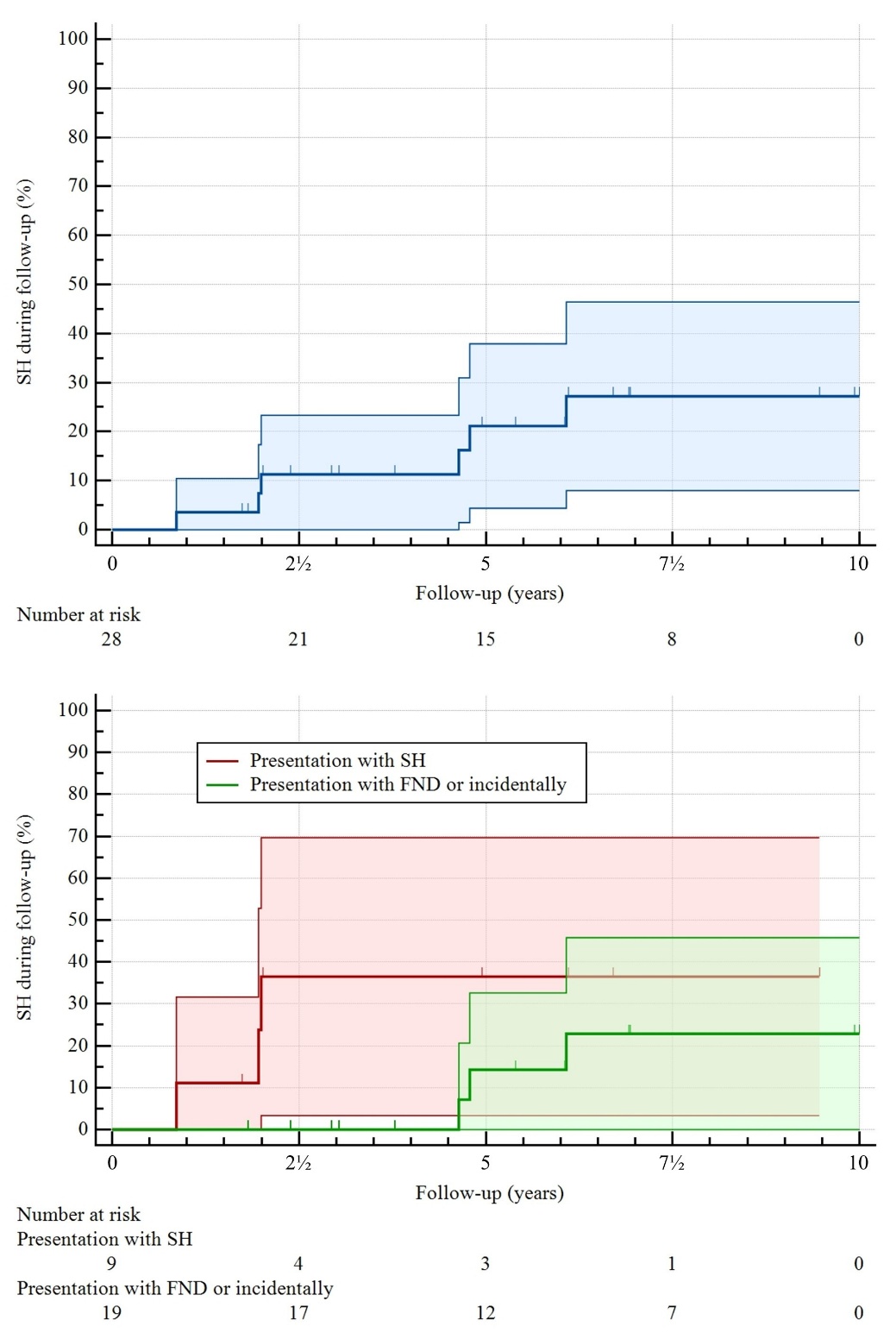


Online Resource 4. Kaplan-Meier analysis for the progression to SH alone during follow-up among all patients in the study population (blue) and stratified by presentation with SH (red) versus presentation with FND or incidentally (green); FND, non-hemorrhagic focal neurological deficit; SH, symptomatic hemorrhage

Online Resource 5. Cumulative 5-year and 10-year rates of SH alone

| **Variable** | **All patients (n=28)** |
| --- | --- |
| Cumulative 5-year rate of SH | 21% (4%-38%) |
| Presentation with SH (n=9) | 37% (3%-70%) |
| Presentation with FND or incidentally (n=19) | 14% (0%-33%) |
| Cumulative 10-year rate of SH | 27% (8%-46%) |
| Presentation with SH (n=9) | 37% (3%-70%) |
| Presentation with FND or incidentally (n=19) | 23% (0%-46%) |

Data are rate (95% CI); CI, confidence interval; FND, non-hemorrhagic focal neurological deficit; SH, symptomatic hemorrhage.

Online Resource 6. Distributions of PROMIS-29 domain T scores stratified by patients who underwent surgical treatment during follow-up and those managed conservatively throughout

| **Domain** | **All patients (n=28)** | **Surgical treatment during follow-up (n=5)** | **Conservative management throughout (n=23)** | **P-value** |
| --- | --- | --- | --- | --- |
| Physical Function | 54.0 (43.0-65.3) | 43.0 (43.0-56.6) | 57.8 (43.0-68.2) | 0.17 |
| Anxiety/Fear | 56.8 (51.2-63.5) | 57.0 (48.7-63.5) | 56.8 (53.1-61.9) | 1.00 |
| Depression/Sadness | 55.9 (48.9-61.4) | 53.6 (43.2-62.4) | 55.9 (48.9-61.4) | 0.86 |
| Fatigue | 55.2 (47.3-62.5) | 47.3 (36.8-53.6) | 55.2 (49.9-64.8) | 0.08 |
| Sleep Disturbance | 50.4 (43.7-58.7) | 49.7 (41.9-57.9) | 50.4 (44.1-58.8) | 0.80 |
| Social Participation | 53.2 (48.1-57.7) | 53.8 (44.1-55.8) | 53.2 (48.1-60.1) | 0.86 |
| Pain Interference | 55.7 (47.5-62.3) | 56.6 (45.1-58.6) | 55.7 (47.5-63.7) | 0.80 |

Data are median (IQR); IQR, interquartile range.

Online Resource 7. Sociodemographic characteristics of all patients in the study population, PROMIS-29 Dutch reference populations, and the 2025 Dutch adult general population

| **Sociodemographic characteristic** | **All patients in the study population** | **Physical function [1]** | **Anxiety/fear [2]** | **Depression/sadness [2]** | **Fatigue [3]** | **Sleep disturbance [4]** | **Social participation [1]** | **Pain interference [1]** | **2025 Dutch adult population [5]** |
| --- | --- | --- | --- | --- | --- | --- | --- | --- | --- |
|  | **n=28** | **n=1310** | **n=1002** | | **n=1006** | | **n=1002** | **n=1052** | **n=15M** |
| Age (mean ±SD) | 47 ±18 | 51 ±17 | 50 ±17 | | 52 ±17 | | 51 ±17 | 52 ±16 | - |
| 18-39 | 36 | 35 | 34 | | 33 | | 32 | 32 | 35 |
| 40-64 | 46 | 42 | 44 | | 44 | | 46 | 45 | 40 |
| ≥65 | 18 | 23 | 21 | | 24 | | 23 | 23 | 25 |
| Sex (male) |  | | | | | | | | |
| Male | 68 | 47 | 48 | | 47 | | 48 | 47 | 50 |
| Female | 32 | 53 | 52 | | 53 | | 52 | 53 | 50 |
| Education |  | | | | | | | | |
| Low | - | 31 | 32 | | 28 | | 29 | 28 | 27 |
| Middle | - | 40 | 40 | | 41 | | 43 | 40 | 37 |
| High | - | 29 | 28 | | 31 | | 28 | 32 | 36 |
| Residence |  | | | | | | | | |
| North | - | 9 | 11 | | 11 | | 10 | 10 | 10 |
| East | - | 21 | 20 | | 20 | | 20 | 21 | 21 |
| South | - | 20 | 21 | | 23 | | 20 | 21 | 21 |
| West | - | 49 | 47 | | 45 | | 50 | 47 | 48 |
| Ethnicity |  | | | | | | | | |
| Native | 89 | 80 | 80 | | 79 | | 77 | 77 | 73 |
| 1^st^ and 2^nd^ generation   western immigrant | 11 | 12 | 13 | | 11 | | 13 | 12 | 11 |
| 1^st^ and 2^nd^ generation   non-west. immigrant |  | 9 | 8 | | 10 | | 10 | 12 | 16 |

Data are percentages (%) unless otherwise specified; M, million; SD, standard deviation.

1. Terwee CB, Roorda LD (2023) Country-specific reference values for PROMIS(®) pain, physical function and participation measures compared to US reference values. Ann Med 55(1):1–11. https://doi.org/10.1080/07853890.2022.2149849.

2. Elsman EBM, Flens G, de Beurs E, Roorda LD, Terwee CB (2022) Towards standardization of measuring anxiety and depression: Differential item functioning for language and Dutch reference values of PROMIS item banks. PLoS One 17(8):e0273287. https://doi.org/10.1371/journal.pone.0273287.

3. Terwee CB, Elsman EB, Roorda LD (2022) Towards standardization of fatigue measurement: Psychometric properties and reference values of the PROMIS Fatigue item bank in the Dutch general population. Research Methods in Medicine & Health Sciences 3(3):86–98. https://doi.org/10.1177/26320843221089628.

4. Terwee CB, van Litsenburg RRL, Elsman EBM, Roorda LD (2023) Psychometric properties and reference values of the Patient-Reported Outcomes Measurement Information System (PROMIS) sleep item banks in the Dutch general population. J Sleep Res 32(2):e13753. https://doi.org/10.1111/jsr.13753.
5. Statistics Netherlands: https://www.cbs.nl.
