# Supplementary material for: Clinical course and patient-reported outcomes in conservatively managed spinal cavernous malformations
Source: J Neurol. 2026 Mar 8;273(3):188. doi: 10.1007/s00415-026-13715-2 (PMC12968117; doi:10.1007/s00415-026-13715-2)
Supplement: Supplementary file 2 — Supplementary file2 (PDF 296 KB) [file 415_2026_13715_MOESM2_ESM.pdf]

NICA registratie vragenlijst over caverneuze malformaties

Database onderzoek naar neurovasculaire aandoeningen

Geachte meneer/mevrouw,

Hierbij vindt u een vragenlijst die onderdeel is van uw deelname aan de NICA registratie. Deze vragenlijst kunt u ook digitaal invullen. Om via een e-mail een link naar de vragenlijst te ontvangen hebben wij uw e-mailadres nodig. Als wij deze nog niet van u in ons bezit hebben willen wij u vragen een e-mail te sturen naar nicaregistratie@amsterdamumc.nl met daarin uw naam en geboortedatum. De e-mail met de link naar de vragenlijst ontvangt u dan binnen enkele dagen. Wij vragen u zo accuraat mogelijk antwoord te geven op de gestelde vragen. Alvast bedankt!

1. Algemene gegevens

1.1 Studienummer: ____ (door onderzoeker ingevuld)

1.2 Geboortedatum: ____ (dd/mm/jjjj)

1.3 Geslacht: ____________ 1.4 Land van herkomst: ______________________

1.5 Lengte: ____________ cm 1.6 Gewicht: ____________ kg

1.7 Datum van invullen: ____ (dd/mm/jjjj)

2. Algemene vragen

2.1 Wat is de naam van uw huisarts en/of huisartsenpraktijk?

____

2.2 Wat is de naam van uw behandelend neuroloog (indien bekend)?

____

2.3 In welk ziekenhuis wordt u opgevolgd of behandeld voor uw caverneuze malformatie?

____

2.4 Heeft u familieleden die ook bekend zijn met caverneuze malformaties?

O nee O ja, namelijk ____ (bv. broer)

2.5 Heeft u genetische testen ondergaan en zo ja was het resultaat een mutatie?

O nee O ja, maar geen mutatie O ja, CCM1 O ja, CCM2 O ja, CCM3

2.6 Gebruikt u vitamine D als supplement?

O nee O ja

2.7 Welke eventuele medicijnen gebruikt u op dit moment?

**Naam (bv. metformine)**	**Sinds (bv. '19)**	**Naam**	**Sinds**
1.		7.	
2.		8.	
3.		9.	
4.		10.	
5.		11.	
6.		12.	

(Als alternatief is het ook mogelijk een medicatielijst van uw apotheek mee te sturen.)

3. Risicofactoren

3.1 Rookt u? O nooit gedaan of ≥half jaar gestopt

O ja of <half jaar gestopt, aantal sigaretten per dag: ________

3.2 Drinkt u alcohol? O nooit of ≥half jaar gestopt

O ja, of <half jaar gestopt, aantal eenheden per week: ______

3.3 Gebruikt u drugs? O nee

O ja, namelijk:____

4. Symptomen

4.1 Heeft u **hersenbloedingen** gehad veroorzaakt door uw caverneuze malformatie(s)?

O nee

O ja, namelijk op:

(dd/mm/jjjj

of schatting)

1.	6.
2.	7.
3.	8.
4.	9.
5.	10.

4.2 Heeft u **epileptische aanvallen** gehad veroorzaakt door uw caverneuze malformatie(s)?

O nee

O ja, namelijk op:

(dd/mm/jjjj

of schatting)

1.	8.
2.	9.
3.	10.
4.	11.
5.	12.
6.	13.
7.	14.

4.3 Heeft u **uitvalsverschijnselen** gehad veroorzaakt door uw caverneuze malformatie(s)?

O nee

O ja, namelijk op:

(dd/mm/jjjj

of schatting)

1.	6.
2.	7.
3.	8.
4.	9.
5.	10.

4.4 Heeft u **hoofdpijn** gehad specifiek veroorzaakt door uw caverneuze malformatie(s) die u bij werk, studie, huishouden of sociaal beperkte, of een bezoek aan een arts vereiste?

O nee

O ja, namelijk op:

(dd/mm/jjjj

of schatting)

1.	7.
2.	8.
3.	9.
4.	10.
5.	11.
6.	12.

5. Behandelingen

Welke eventuele behandelingen heeft u voor uw caverneuze malformaties(s) gekregen?

(Bijvoorbeeld een operatie en/of bestraling, of een specifiek medicijn als behandeling.)

**Soort behandeling**	**Datum (dd/mm/jjjj)**
1.	
2.	
3.	
4.	
5.	
6.	
7.	
8.	
9.	
10.	

<u>U bent over de helft van de vragenlijst!</u>

Zet bij iedere groep in de lijst hieronder een kruisje in het hokje dat het best past bij uw gezondheid VANDAAG.

MOBILITEIT

Ik heb geen problemen met lopen ❑
Ik heb een beetje problemen met lopen ❑
Ik heb matige problemen met lopen ❑
Ik heb ernstige problemen met lopen ❑
Ik ben niet in staat om te lopen ❑

ZELFZORG

Ik heb geen problemen met mijzelf wassen of aankleden ❑
Ik heb een beetje problemen met mijzelf wassen of aankleden ❑
Ik heb matige problemen met mijzelf wassen of aankleden ❑
Ik heb ernstige problemen met mijzelf wassen of aankleden ❑
Ik ben niet in staat mijzelf te wassen of aan te kleden ❑

DAGELIJKSE ACTIVITEITEN *(bijv. werk, studie, huishouden, gezins- en vrijetijdsactiviteiten)*

Ik heb geen problemen met mijn dagelijkse activiteiten ❑
Ik heb een beetje problemen met mijn dagelijkse activiteiten ❑
Ik heb matige problemen met mijn dagelijkse activiteiten ❑
Ik heb ernstige problemen met mijn dagelijkse activiteiten ❑
Ik ben niet in staat mijn dagelijkse activiteiten uit te voeren ❑

PIJN/ONGEMAK

Ik heb geen pijn of ongemak ❑
Ik heb een beetje pijn of ongemak ❑
Ik heb matige pijn of ongemak ❑
Ik heb ernstige pijn of ongemak ❑
Ik heb extreme pijn of ongemak ❑

ANGST/SOMBERHEID

Ik ben niet angstig of somber ❑
Ik ben een beetje angstig of somber ❑
Ik ben matig angstig of somber ❑
Ik ben erg angstig of somber ❑
Ik ben extreem angstig of somber ❑

Netherlands (Dutch) © 2009 EuroQol Group. EQ-5D™ is a trade mark of the EuroQol Group

- We willen weten hoe goed of slecht uw gezondheid VANDAAG is.
- Deze meetschaal loopt van 0 tot 100.
- 100 staat voor de <u>beste</u> gezondheid die u zich kunt voorstellen.
  0 staat voor de <u>slechtste</u> gezondheid die u zich kunt voorstellen.
- Markeer een X op de meetschaal om aan te geven hoe uw gezondheid VANDAAG is.
- Noteer het getal waarbij u de X heeft geplaatst in onderstaand vakje.

UW GEZONDHEID VANDAAG = ☐

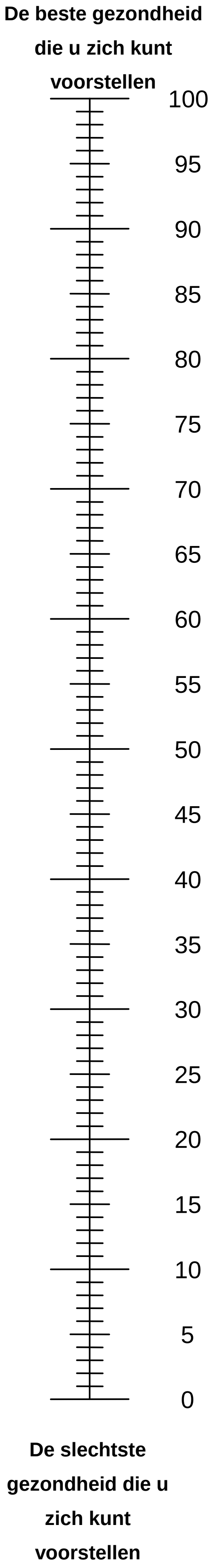

Netherlands (Dutch) © 2009 EuroQol Group. EQ-5D™ is a trade mark of the EuroQol Group

PROMIS-29 Profile v2.1

Geef een reactie op elke vraag of uitspraak door per rij één hokje aan te kruisen.

	**Lichamelijk functioneren**	**Zonder moeite**	**Met een beetje moeite**	**Met enige moeite**	**Met veel moeite**	**Kan het niet**
PFA11	Kunt u klusjes doen zoals stofzuigen of in de tuin werken?	☐ 5	☐ 4	☐ 3	☐ 2	☐ 1
PFA21	Kunt u in een normaal tempo trappen op- en afgaan?	☐ 5	☐ 4	☐ 3	☐ 2	☐ 1
PFA23	Kunt u een wandeling van ten minste 15 minuten maken?	☐ 5	☐ 4	☐ 3	☐ 2	☐ 1
PFA53	Kunt u boodschappen doen en winkelen?	☐ 5	☐ 4	☐ 3	☐ 2	☐ 1

	**Angst**<br>**Geef a.u.b. antwoord voor de afgelopen 7 dagen.**	**Nooit**	**Zelden**	**Soms**	**Vaak**	**Altijd**
EDANX01	Ik voelde me angstig.	☐ 1	☐ 2	☐ 3	☐ 4	☐ 5
EDANX40	Ik vond het moeilijk om me op iets anders dan mijn angst en bezorgdheid te concentreren.	☐ 1	☐ 2	☐ 3	☐ 4	☐ 5
EDANX41	Mijn zorgen waren me te veel.	☐ 1	☐ 2	☐ 3	☐ 4	☐ 5
EDANX53	Ik voelde me slecht op mijn gemak.	☐ 1	☐ 2	☐ 3	☐ 4	☐ 5

	**Depressie**<br>**Geef a.u.b. antwoord voor de afgelopen 7 dagen.**	**Nooit**	**Zelden**	**Soms**	**Vaak**	**Altijd**
EDDEP04	Ik voelde me alsof ik niets waard was.	☐ 1	☐ 2	☐ 3	☐ 4	☐ 5
EDDEP06	Ik voelde me hulpeloos.	☐ 1	☐ 2	☐ 3	☐ 4	☐ 5
EDDEP29	Ik voelde me depressief.	☐ 1	☐ 2	☐ 3	☐ 4	☐ 5
EDDEP41	Ik voelde me zonder hoop.	☐ 1	☐ 2	☐ 3	☐ 4	☐ 5

© 2008-2018 PROMIS Health Organization (PHO)

Vermoeidheid

	Geef a.u.b. antwoord voor de afgelopen 7 dagen.	Helemaal niet	Een beetje	Enigszins	In vrij hoge mate	In zeer hoge mate
HI7	Ik heb last van vermoeidheid.	☐ 1	☐ 2	☐ 3	☐ 4	☐ 5
AN3	Het kost me moeite om met dingen te <u>beginnen</u> omdat ik zo moe ben.	☐ 1	☐ 2	☐ 3	☐ 4	☐ 5

		Helemaal niet	Een beetje	Enigszins	Behoorlijk	Heel erg
FATEXP41	Hoe afgepeigerd voelde u zich gemiddeld genomen?	☐ 1	☐ 2	☐ 3	☐ 4	☐ 5
FATEXP40	Hoe vermoeid was u gemiddeld genomen?	☐ 1	☐ 2	☐ 3	☐ 4	☐ 5

Slaapstoornissen

	Geef a.u.b. antwoord voor de afgelopen 7 dagen.	Heel slecht	Slecht	Redelijk	Goed	Heel goed
Sleep109	De kwaliteit van mijn slaap was…	☐ 5	☐ 4	☐ 3	☐ 2	☐ 1

	Geef a.u.b. antwoord voor de afgelopen 7 dagen.	Helemaal niet	Een beetje	Enigszins	Behoorlijk	Heel erg
Sleep116	Mijn slaap was verkwikkend.	☐ 5	☐ 4	☐ 3	☐ 2	☐ 1
Sleep20	Ik had een slaapprobleem.	☐ 1	☐ 2	☐ 3	☐ 4	☐ 5
Sleep44	Ik had moeite met in slaap vallen.	☐ 1	☐ 2	☐ 3	☐ 4	☐ 5

Vermogen om een aandeel te hebben in sociale rollen en activiteiten

		Nooit	Zelden	Soms	Meestal	Altijd
SRPPER11_CaPS	Ik heb moeite om al mijn gewone vrijetijdsactiviteiten met anderen te doen.	☐ 5	☐ 4	☐ 3	☐ 2	☐ 1
SRPPER18_CaPS	Ik heb moeite om alle gezins-/familieactiviteiten te doen die ik wil doen.	☐ 5	☐ 4	☐ 3	☐ 2	☐ 1
SRPPER23_CaPS	Ik heb moeite om al mijn gewone werk (inclusief werk thuis) te doen.	☐ 5	☐ 4	☐ 3	☐ 2	☐ 1
SRPPER46_CaPS	Ik heb moeite om alle activiteiten met vrienden te doen die ik wil doen.	☐ 5	☐ 4	☐ 3	☐ 2	☐ 1

© 2008-2018 PROMIS Health Organization (PHO)

Belemmeringen door pijn

Geef a.u.b. antwoord voor de afgelopen 7 dagen.

		Helemaal niet	Een beetje	Enigszins	Behoorlijk	Heel erg
PAININ9	In welke mate belemmerde de pijn u bij uw dagelijkse activiteiten?	☐ 1	☐ 2	☐ 3	☐ 4	☐ 5
PAININ22	In welke mate belemmerde de pijn u bij werk in en om het huis?	☐ 1	☐ 2	☐ 3	☐ 4	☐ 5
PAININ31	In welke mate belemmerde de pijn uw vermogen om deel te nemen aan sociale activiteiten?	☐ 1	☐ 2	☐ 3	☐ 4	☐ 5
PAININ34	In welke mate belemmerde de pijn u bij uw huishoudelijke klusjes?	☐ 1	☐ 2	☐ 3	☐ 4	☐ 5

Pijnintensiteit

Geef a.u.b. antwoord voor de afgelopen 7 dagen.

Global07	Hoe zou u gemiddeld uw pijn beoordelen?	☐ 0 **Geen pijn**	☐ 1	☐ 2	☐ 3	☐ 4	☐ 5	☐ 6	☐ 7	☐ 8	☐ 9	☐ 10 **Ergst denkbare pijn**

© 2008-2018 PROMIS Health Organization (PHO)

Dit is het einde van de vragenlijst.

Wilt u zo vriendelijk zijn na te gaan of alle vragen zijn beantwoord?

Hartelijk dank voor uw deelname.
